# Combinational Inhibition of P-Glycoprotein-Mediated Etoposide Transport by Zosuquidar and Polysorbate 20

**DOI:** 10.3390/pharmaceutics15010283

**Published:** 2023-01-14

**Authors:** Rasmus Blaaholm Nielsen, René Holm, Ils Pijpers, Jan Snoeys, Ulla Gro Nielsen, Carsten Uhd Nielsen

**Affiliations:** 1 Department of Physics, Chemistry and Pharmacy, University of Southern Denmark, Campusvej 55, DK-5230 Odense, Denmark; 2 Bioanalysis Discovery & Development Sciences, Janssen R & D, Johnson & Johnson, Turnhoutseweg 30, BE-2340 Beerse, Belgium; 3 Drug Metabolism and Pharmacokinetics, Janssen R & D, Johnson & Johnson, Turnhoutseweg 30, BE-2340 Beerse, Belgium

**Keywords:** P-glycoprotein, etoposide, zosuquidar, polysorbate 20, efflux transport, oral absorption

## Abstract

P-glycoprotein (P-gp) limits the oral absorption of drug substances. Potent small molecule P-gp inhibitors (e.g., zosuquidar) and nonionic surfactants (e.g., polysorbate 20) inhibit P-gp by proposedly different mechanisms. Therefore, it was hypothesised that a combination of zosuquidar and polysorbate 20 may potentiate inhibition of P-gp-mediated efflux. P-gp inhibition by zosuquidar and polysorbate 20 in combination was assessed in a calcein-AM assay and in a transcellular etoposide permeability study in MDCKII-MDR1 and Caco-2 cells. Furthermore, solutions of etoposide, zosuquidar, and polysorbate 20 were orally administered to Sprague Dawley rats. Zosuquidar elicited a high level of nonspecific adsorption to various labware, which significantly affected the outcomes of the in vitro studies. Still, at certain zosuquidar and polysorbate 20 concentrations, additive P-gp inhibition was observed in vitro. In vivo, however, oral etoposide bioavailability decreased by coadministration of both zosuquidar and polysorbate 20 when compared to coadministration of etoposide with zosuquidar alone. For future formulation development, the present study provided important and novel knowledge about nonspecific zosuquidar adsorption, as well as insights into combinational P-gp inhibition by a third-generation P-gp inhibitor and a P-gp-inhibiting nonionic surfactant.

## 1. Introduction

P-glycoprotein (P-gp) limits oral absorption of drug substances, such as etoposide, and P-gp inhibitors have been developed to negate P-gp-mediated multidrug resistance in cancer and to increase oral bioavailability [1,2]. Four generations of P-gp inhibitors have been developed thus far (reviewed by Palmeira et al. [3]), yet none of them have reached the market alone or in combination with another compound [4]. Therefore, there is a need to develop drug formulations that can increase the intestinal absorption and oral bioavailability of P-gp substrates. The third-generation inhibitor zosuquidar has been used to increase P-gp-limited oral absorption of etoposide [5]. Likewise, certain nonionic surfactants such as polysorbate 20, polysorbate 80, Cremophor^®^ EL, and Pluronic^®^ P85 also inhibit P-gp-mediated transport in vitro as well as in vivo, increasing the oral absorption of etoposide when coadministered together (reviewed by Al-Ali et al. [6]). Etoposide is useful as a model drug for P-gp substrates, as its bioavailability is limited by P-gp, which was exemplified by increased oral bioavailability in *mdr1a* knockout rats [7]. Moreover, bioanalytical methods are available [5,7,8], with one drawback to using etoposide being its cytotoxic properties [9]. Several formulation approaches have been attempted to increase oral etoposide absorption by either inhibiting P-gp with inhibitors or increasing the solubility of etoposide in the intestine to saturate P-gp. The formulations were, e.g., an oral solution containing surfactants [7], oral supersaturated solutions containing surfactants [10], and amorphous solid dispersions of etoposide:copovidon:eudragit L-100 [8]. There is, however, still a need to develop formulations that can increase the bioavailability of P-gp substrates. The present work presents a novel approach where formulations with multiple P-gp inhibitors are applied, thereby possibly reducing the dose required of the individual inhibitors. We here investigated combining a nonionic surfactant, polysorbate 20, with a small molecular inhibitor, zosuquidar. While the binding mechanism of zosuquidar to the central binding pocket of P-gp is well described, with two zosuquidar molecules competitively binding to cause inhibition of substrate transport [11,12,13], the ability of nonionic surfactants to inhibit P-gp appears more complex. The inhibitory mechanism for polysorbate 20 has been suggested to be mediated by direct binding to the transporter [14], alteration of membrane fluidity [15], and depletion of intracellular ATP [16], as well as combinations hereof (reviewed by Al-Ali et al. [6]). Recently, it was reported that polysorbate 20 binds to P-gp at the interface of the outer leaflet without entering the inner leaflet and moves along the protein to also occupy the central binding cavity [17]. Moreover, polysorbate increases the energy required for the protein to return to the outward facing conformation, where the protein is available for substrate binding [17]. Therefore, it may be hypothesised that polysorbate 20 could act to increase the residence time of zosuquidar in the binding pocket of P-gp, and thereby increase the time, where P-gp is in an occluded state. This could increase the effect of zosuquidar and increase oral absorption of a P-gp drug substrate, such as etoposide. To the best of our knowledge, no studies have combined a specific small molecule P-gp inhibitor and a P-gp inhibiting nonionic surfactant to increase the oral absorption of a P-gp substrate. As zosuquidar and polysorbate 20 inhibit P-gp by different mechanisms, we hypothesised that zosuquidar and polysorbate 20 may elicit synergistic inhibitory effects on P-gp in vitro and in vivo, which could be used for enabling formulations of P-gp substrates. Therefore, the present study aimed to assess the combinational effect of zosuquidar and polysorbate 20 on P-gp-mediated etoposide transport in vitro. It is desired to reduce, refine, and even replace animal studies; however, for drug transporters and formulations aimed at modifying their activity, there are currently not much data to support the exclusive use of in vitro models, even though 3D cell culture models are emerging [18]. Therefore, more in vitro–in vivo data are needed before animal studies can be replaced with advanced in vitro models for predicting the in vivo performance of enabling formulations. Therefore, the aim was also to assess oral etoposide absorption in Sprague Dawley rats after coadministration with combinations of zosuquidar and polysorbate 20. During the experimental work for the present publication and a previous publication [5], it was discovered that zosuquidar unexpectedly elicited low solubility and nonspecific adsorption to labware surfaces. Polysorbate 20 may affect the solubility or adsorption behaviour of zosuquidar. Thus, the present study also aimed to systematically characterise zosuquidar solubility and nonspecific adsorption of zosuquidar in the absence or presence of polysorbate 20, as well as in commonly applied buffers and organic solvents.

## 2. Materials and Methods

A water purification system was applied to obtain ultrapure water (Millipore, Boston, MA, USA). Fetal bovine serum was from VWR (Biowest, Kansas City, PA, USA). ^3^H-mannitol was from Perkin Elmer (Hopkinton, MA, USA). Etoposide and zosuquidar 3HCl was obtained from Medkoo (Morrisville, NC, USA). Unless explicitly stated, ‘zosuquidar’ always refers to the free molecule without the HCl salt. Simulated fasted intestinal fluid (FaSSIF-V2) powder was obtained from Biorelevant (London, UK). All other chemicals were from Merck (Readington Township, NJ, USA) and were applied in the received quality, which was analytical-grade or higher.

### 2.1. Determination of Zosuquidar’s pK_a_ Values

Determination of pK_a_ values was performed using a Sirius T3 titrator (Pion, East Sussex, UK), measuring the UV-VIS spectrum during titration. Experiments were conducted at 25 °C. The ionic strength was kept constant by using water containing 0.15 M potassium chloride. The pK_a_ of zosuquidar was determined using DMSO as a cosolvent and extrapolated to 0% cosolvent by using the Yasuda–Shedlovsky method [19,20]. Data were processed using the accompanying Sirius software (version 2.0.0.0, Pion, East Sussex, UK).

### 2.2. Zosuquidar Solubility

Zosuquidar suspensions containing approximately 0.15 mg/mL zosuquidar were prepared in 10 mM HEPES HBSS pH 7.4 with or without 5, 10, or 50 µM polysorbate 20, 1% (*v*/*v*) methanol, or 1% (*v*/*v*) DMSO in 1.5 mL microtubes (Sarstedt, Germany), *n* = 4. Samples were shaken on a vertical rotator (10 rpm, PTR-60, Grant Instruments, Cambridge, UK) for approximately 72 h at ambient temperature. The suspensions were then centrifuged at ambient temperature (15,000 RCF, 10 min, Multifuge X1R, Thermo Fisher Scientific, Boston, MA, USA), and a sample of the supernatant was transferred and diluted 1:1 with mobile phase in a 2 mL glass HPLC vial and analysed by fluorescence-coupled HPLC (HPLC-FL).

### 2.3. Nonspecific Zosuquidar Adsorption

Preliminary studies had shown that zosuquidar tended to adsorb to various common glass- or plasticware. Generally, when zosuquidar was diluted in aqueous solutions, less zosuquidar than expected was present according to HPLC-FL quantitation, when compared to the corresponding dilutions in water–organic solvent mixtures. Zosuquidar adsorption to the surface of 2 mL glass HPLC vials was therefore investigated in various aqueous media, as well as in aqueous–organic solvent mixtures. Zosuquidar 3HCl was weighed and dissolved in methanol or DMSO to achieve stock solutions of 10 or 20 mM zosuquidar. These were further diluted in methanol or DMSO to 100 µM. For adsorption experiments, solutions of 0.5–49.5% *v*/*v* methanol or DMSO in HEPES HBSS pH 7.4 were prepared, along with HEPES HBSS pH 7.4 supplemented with 0.10, 1.0, 2.5, 5.0, 10, or 50 µM polysorbate 20 or 0.05% bovine serum albumin, as well as 10 mM MES HBSS pH 6.5, 10 mM ammonium acetate pH 4.5, 19 mM maleic acid buffer pH 6.5, and FaSSIF-V2. A total of 995 µL of the desired medium was transferred to a 2 mL HPLC vial, and 5 µL 100 µM zosuquidar in either methanol or DMSO was then added for a final apparent concentration of 500 nM zosuquidar and 0.5% methanol or DMSO. Furthermore, pure methanol, HEPES HBSS pH 7.4, and 100 µM zosuquidar in methanol was combined to a total volume of 1000 µL in HPLC vials to achieve concentrations of 0.75, 2.5, 7.5, and 25 µM zosuquidar in 25% *v*/*v* methanol in HEPES HBSS pH 7.4. Control samples were prepared by addition of 5 µL 100 µM zosuquidar in methanol to 995 µL zosuquidar mobile phase, which were treated like the other samples. The vials were capped and vortexed for 1 min and equilibrated at ambient temperature for approximately 24 h before HPLC-FL analysis. The peak area of the samples was normalised to the peak area of control samples, which were considered to elicit 0% adsorption. Furthermore, a simple control experiment was conducted to ensure that the observed disappearance of zosuquidar was caused by adsorption to surfaces and not by alteration of the HPLC-FL signal induced by the solvent. Two vials (A1 and B1, *n* = 3) were filled with HEPES HBSS pH 7.4 and spiked with a concentrated zosuquidar solution in methanol to obtain an apparent concentration of 500 nM zosuquidar and 0.5% methanol (five-fold below equilibrium solubility). Both vials were equilibrated at room temperature for at least 24 h. A sample from A1 was transferred to a new vial, A2, and diluted 1:1 in mobile phase for an apparent concentration of 250 nM zosuquidar. In vial B1, the solution was directly diluted 1:1 in mobile phase in the same vial, likewise for an apparent concentration of 250 nM zosuquidar. Thus, the resulting solutions consisted of the same solvent (HEPES HBSS pH 7.4: mobile phase) and would have the same zosuquidar concentration if zosuquidar acted ideally in solution. The solutions in both vials were then analysed directly by HPLC-FL.

### 2.4. Cell Cultivation

Caco-2 cells were obtained from Deutsche Sammlung von Mikroorganismen und Zellkulturen (DSMZ) and MDCKII-MDR1 cells were obtained from the laboratory of Prof. Piet Borst (Netherlands Cancer Institute, Amsterdam, The Netherlands). Cells were kept in Dulbecco’s modified eagle medium supplied with 10% foetal bovine serum, penicillin (100 U/mL), streptomycin (100 µg/mL), L-glutamine (2 mM), and non-essential amino acids (1×) under atmospheric air supplied with 5% CO_2_ and 95% relative humidity (Heracell 150i incubator, Thermo Fisher Scientific, Boston, MA, USA). Culture medium was changed three times per week.

For transcellular permeability studies, Caco-2 and MDCKII-MDR1 cells were seeded at a concentration of 1.0 or 1.6 × 10^5^ cells per filter, respectively, on 1.12 cm^2^ polycarbonate inserts (0.4 µM pore size, Corning, Corning, NY, USA). Experiments were performed 14 or 3 days after seeding, respectively. For calcein-AM assays, MDCKII-MDR1 cells were seeded at a density of 6.4 × 10^4^ cells per well in black, clear bottom, 96-well plates.

### 2.5. Transcellular Etoposide Permeability across Cell Monolayers

Transcellular permeability of etoposide was assessed in three series: (1) in Caco-2 cells, (2) in MDCKII-MDR1 cells, and (3) in Caco-2 cells again. See Table 1 for overview. All were carried out in four independent cell passages. Transcellular permeability studies were performed in HEPES HBSS pH 7.4 and were assessed in the basolateral (B) to apical (A) and A–B direction for series (1) and (3) and in the B–A direction for series (2). Donor solutions contained 50 µM etoposide. For polysorbate 20 treatments, polysorbate 20 was always only present in the apical compartment, and zosuquidar was present both apically and basolaterally. Test solutions were either prepared by conventional serial dilution for series (1)–(2) or by a spike method for series (3). The spike method was described previously [5]. Briefly, the spike method consisted of preparing 100-times-concentrated zosuquidar and/or polysorbate 20 solutions in pure methanol and spiking these solutions into the donor and receiver buffers in the relevant compartments of the Transwell plate and inserts at the start of the experiment. As will be shown in the present publication, conventional serial dilution will not lead to the desired concentration of free zosuquidar in solution, and therefore, zosuquidar concentrations in solutions prepared by conventional serial dilution are reported as apparent concentrations.

Before the transport experiment, growth medium was removed from the cells by vacuum suction (Vacusip, Integra Biosciences, NH, USA), followed by a 10 min equilibration in HEPES HBSS pH 7.4 on a Talboys 1000 MP incubating microplate shaker (Troemner, NJ, USA, 220 rpm, 37 °C). Then, the buffer was removed, and donor and acceptor solutions were added, with a total of 500 and 1000 µL to the A and B compartment, respectively. Samples of 50 µL were taken from the receiver compartment at 15, 30, 60, 90, and 120 min and diluted 1:1 in etoposide mobile phase. At 120 min, a sample was taken from the donor compartment and diluted in mobile phase. Samples were analysed by HPLC-FL, and for series (2) and (3), 50 µL sample was transferred to a black, clear-bottom 96-well plate for lucifer yellow quantification.

#### 2.5.1. Assessment of Cell Monolayer Integrity

For all series, cell layer integrity was assessed by measurements of transepithelial electrical resistance (TEER) at room temperature. In Caco-2 cells (series 1 and 3), initial TEER values ranged from 305–605 and 557–747 Ω × cm^2^ and on average dropped by 33 or 41% during the experimental time. No single filter displayed a drop in TEER of more than 68 or 65% during the experiment, respectively. For series 2 (MDCKII-MDR1), initial TEER values ranged from 71–95 Ω × cm^2^ and on average dropped by 19% during the experiment, and no single filter displayed a drop of more than 34%. These changes were consistent with our previous studies [5,21], and cell monolayers were considered intact.

For series (1), barrier properties were further assessed by measuring the paracellular permeability of ^3^H-mannitol after the etoposide permeability experiment. Each apical compartment was spiked with 10 µL 0.05 µCi/µL and 17.2 Ci/mmol ^3^H-mannitol, and 50 µL samples were taken from the basolateral compartment at 10, 20, and 30 min after addition, and a donor sample was taken from the apical compartment at 30 min. ^3^H-mannitol permeability was in the range 0.31–1.4 × 10^−6^ cm/s and none of the treatments affected ^3^H-mannitol permeability. For (2) and (3), passive permeability was assessed by supplementation of 100 µM lucifer yellow to all donor solutions. After HPLC-FL analysis, acceptor and donor samples were transferred to a black, clear-bottom 96-well plate and quantified by a Fluostar Omega plate reader (BMG Labtech, Germany) by a previously reported method [5]. Lucifer yellow permeability was in the range 0.39–1.2 and 0.15–1.1 × 10^−6^ cm/s, respectively, and no treatment affected lucifer yellow P_app_. Collectively, all cell monolayers were considered intact.

#### 2.5.2. Data Analysis

Etoposide, lucifer yellow, and ^3^H-mannitol flux across cell monolayers were calculated individually for each passage by linear regression of the steady-state part (30–120 min) of the molar amount accumulated in the receiver chamber per filter area vs. time. P_app_ was calculated by dividing the flux with the measured donor concentration. Etoposide P_app_ was plotted against apparent zosuquidar concentration and the IC_50_ value was estimated by regression performed in Prism 8.4 (Graphpad, San Diego, CA, USA):

(1) $$Y=Bottom+\frac{Top-Bottom}{1+{\left(\frac{IC_{50}}{X}\right)}^{Hill\;Slope}}$$

where Y is etoposide P_app_; Bottom and Top are the low and high etoposide P_app_ plateaus, respectively; IC_50_ is the zosuquidar concentration at the midway point between low and high P_app_ plateaus; and the Hill Slope is the Hill coefficient, determining the slope of the dose–response curve.

### 2.6. Calcein-AM Assay

Before the experiment, growth medium was removed by vacuum suction (Vacusip, Integra Biosciences, NH, USA), 100 µL HEPES HBSS pH 7.4 was added to each well with a multichannel pipette, and the cells were incubated on a Talboys 1000 MP incubating microplate shaker (Troemner, NJ, USA, 220 rpm, 37 °C).

To demonstrate the effect of making conventional serial dilutions of zosuquidar vs. a spike method, we designed a protocol for a calcein-AM assay where test solutions were prepared and applied by two different methods: (i) Zosuquidar solutions were prepared by serial dilution of zosuquidar in HEPES HBSS pH 7.4 with or without supplementation of 0.1, 1.0, 2.5, or 5.0 µM polysorbate 20. Solutions were prepared by preparation of 10 µM zosuquidar solutions in HEPES HBSS pH 7.4 with or without 0.1, 1.0, 2.5, or 5.0 µM polysorbate 20. The 10 µM zosuquidar solutions were added to the top row of a 96-well clear plate, and HEPES HBSS pH 7.4 with or without corresponding polysorbate 20 concentrations were added to all wells below, and the 10 µM zosuquidar solutions were then diluted serially 1:3 in the 96-well plate with a multichannel pipette along the columns of the plate. A total of 170 µL of the test solutions could then be transferred from the 96-well plate to the cell-containing plate at the start of preincubation. (ii) For the spike method, 162 µL HEPES HBSS pH 7.4 was transferred to each cell-containing well with a multichannel pipette, and 8 µL of a 25-times-concentrated zosuquidar solution in 25% methanol was then added individually to the relevant wells with a serological pipette for preincubation. Wells containing HEPES HBSS pH 7.4 only served as a positive control. The cells were preincubated with test solutions (serial and spike) for 10 min on a microplate shaker (37 °C, 220 rpm). To start the experiment, 30 µL 33.3 µM calcein-AM in HEPES HBSS pH 7.4 was added to each well with a multichannel pipette, and the plate was stirred for 1 min on a microplate shaker (37 °C, 220 rpm) before being transferred to a Fluostar Omega plate reader. A time-resolved fluorescence intensity program of approximately 1 h was run at 37 °C with 60 cycles of 63 s, top optics set at 483-14 excitation and 530-30 emission, 3 flashes per well, and a gain setting of 792.

One column of wells was kept without cells to measure background fluorescence signals from the applied buffers and compounds. No buffers or compounds in the applied concentrations elicited any fluorescence signals, and the acquired signals were subtracted from the remaining fluorescence signals as background. After background subtraction, P-gp activity was estimated by linear regression of the linear part of the fluorescence intensity vs. time plot followed by normalisation to the control column with full P-gp activity:

(2) $$P-gp\;activity=\frac{1}{\frac{slope_{test}}{slope_{control}}}\times 100\%$$

P-gp activity was then plotted against apparent zosuquidar concentration and was fitted to Equation (2), where Y is P-gp activity; X is apparent zosuquidar concentration; Bottom and Top are the low and high P-gp activity plateaus, respectively; IC_50_ is the zosuquidar concentration at half-maximal P-gp activity; and Hill Slope is the Hill coefficient, determining the slope of the dose–response curve.

### 2.7. Etoposide and Zosuquidar Quantification by HPLC-FL

Zosuquidar and etoposide in samples from adsorption, solubility, and transcellular permeability experiments were quantified by a previously reported method [5], using an HPLC-FL (Shimadzu, Kyoto, Japan). Etoposide and zosuquidar were detected at 230/330 and 240/415 nm excitation/emission, respectively. Mobile phase consisted of 38:61.5:0.5 methanol:ultrapure water:acetic acid or of 57:43 methanol:35 mM ammonium acetate pH 4.5 for etoposide or zosuquidar, respectively. A reversed-phase column kept at 40 °C was applied (XBridge C18 2.5 µM, 2.1 × 30 mm, Waters, Milford, MA, USA) with a 0.3 mL/min flow rate. The retention time was 2.0 and 2.4 min, and the lower limits of quantification (LOQ) were 35 and 8 nM for etoposide and zosuquidar, respectively.

### 2.8. Pharmacokinetic Study in Sprague Dawley Rats

The study was conducted according to the European Convention for the Protection of Vertebrate Animals used for Experimental and other Scientific Purposes (ETS no. 123) and Belgian law controlling the experiments on animals (Royal Decree of 29 May 2013 for the protection of laboratory animals). Animals were allowed minimum five days to acclimatise upon arrival from the supplier, and they were housed in polysulphone cages (58 × 52 × 20 cm) in groups of six provided with wooden sticks for enrichment. Animals had free access to Safe 04 Maintenance Diet (Safe, Rosenberg, Germany) and water during acclimatisation and during the study.

#### 2.8.1. Study Design, Dosing, and Sampling

Male Sprague Dawley rats (Charles River, Dortmund, Germany) weighing 293–358 g were randomly assigned into six groups of six animals. Formulations were prepared as clear solutions in 40% *v*/*v* ethanol–water and 10 mL/kg was administered to each animal by oral gavage. All animals received 20 mg/kg etoposide supplemented with zosuquidar and/or polysorbate 20: group (A) + 1% polysorbate 20, (B) + 1.0% polysorbate 20 + 0.63 mg/kg zosuquidar, (C) + 5.0% polysorbate 20, (D) + 5.0% polysorbate 20 + 2.0 mg/kg zosuquidar, (E) + 5.0% polysorbate 20 + 6.3 mg/kg zosuquidar, (F) + 10.0% polysorbate 20 + 6.3 mg/kg zosuquidar. Etoposide, zosuquidar, and polysorbate 20 were all freely dissolved in the 40% ethanol–water mixture upon administration. It was previously shown that corresponding formulations of etoposide and zosuquidar in 40% ethanol did not precipitate upon dilution in simulated rat intestinal fluid [5]. Plasma samples were taken by tail vein puncture with a needle followed by collection in 32–64 µL micro haematocrit tubes (Vitrex Medical, Herlev, Denmark) at times 15, 30, and 45 min and 1, 2, 3, 4, and 6 h. Samples were centrifuged, and plasma was transferred into 10 µL end-to-end pipettes (Vitrex Medical, Herlev, Denmark) and immediately frozen at −80 °C until bioanalysis. Immediately after the study, animals were euthanised according to the principles of euthanasia stated in the AVMA Guidelines for the Euthanasia of Animals (American Veterinary Medical Association, 2020). Plasma samples were analysed by a previously described qualified LC-MS/MS method [5].

#### 2.8.2. Data Analysis

Pharmacokinetic parameters were calculated individually for each animal subject and pooled for statistical analysis. The plasma concentration at 15 min (C_15min_) was reported as an average of the plasma concentration of the six animals in each group. The maximal plasma concentration (C_max_) and the corresponding time at C_max_ (t_max_) was identified for each animal, and C_max_ was reported as mean ± SEM, while t_max_ was reported as median and 1st and 3rd quartile. Area under the curve from 0–6 h (AUC_0–6_) was calculated in GraphPad Prism 8.4, and absolute bioavailability was calculated based on dose-corrected AUC_0–6_ reported in a previous study [5]. Elimination rate constants (k_e_) were calculated by linear regression of the linear part of the ln(plasma concentration) versus time profiles, and plasma half-life (t_½_) was calculated from k_e_:

(3) $$t_{½}=\frac{\ln\;(2)}{k_{e}}$$

## 3. Results and Discussion

### 3.1. Zosuquidar pK_a_ and Solubility

**Figure 1:**
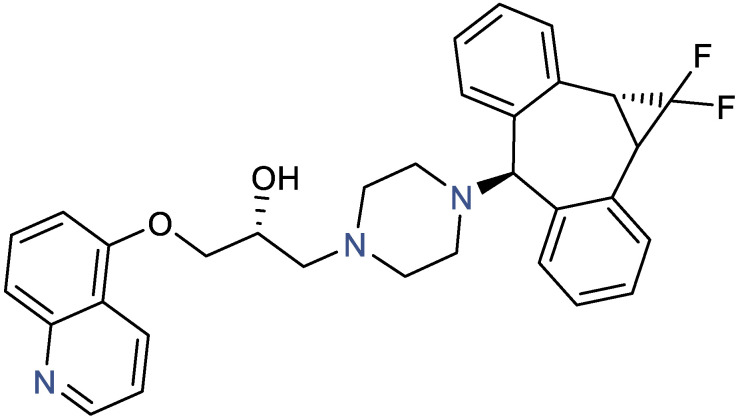
Zosuquidar structure. Experimentally determined pK_a_ values of 1.5, 4.8, and 7.9 represents protonation of the three N-atoms (blue). Drawn in ChemDraw Professional v. 16.0.1.4 (Perkin Elmer Informatics, Hopkinton, MA, USA).

The two lowest pK_a_ values of zosuquidar (pK_a1_ and pK_a2_) were determined in pure water to 1.5 ± 0.4 and 4.78 ± 0.03, respectively. pK_a3_ could not be determined in water due to precipitation of the neutral complex. Both pK_a2_ and pK_a3_ were determined by applying DMSO as a cosolvent followed by extrapolation to 0% DMSO, using the Yasuda–Shedlovsky method. With this method, pK_a2_ and pK_a3_ were 4.83 ± 0.03 and 7.94 ± 0.01, respectively, while pK_a1_ could not be determined. These three pK_a_ values likely represent the protonation of the three N-atoms in the chemical structure of zosuquidar (Figure 1). Thus, at pH values between 4.8 and 7.9, the dominant form of zosuquidar carries one protonated N-atom and thereby one positive charge, and the degree of protonation and positive charge increases with decreasing pH value. To the best of our knowledge, experimental logP- or logD values are not available in literature, but the predicted logP of zosuquidar has been reported to be in the range from 4.8 to 5.2 [22], meaning that the neutral form of zosuquidar is highly hydrophobic. Considering that approx. 90% of zosuquidar will carry one positive charge at pH 7, the physiologically relevant logD at pH 7 will likely be lower, and logD will decrease with decreasing pH.

**Figure 2:**
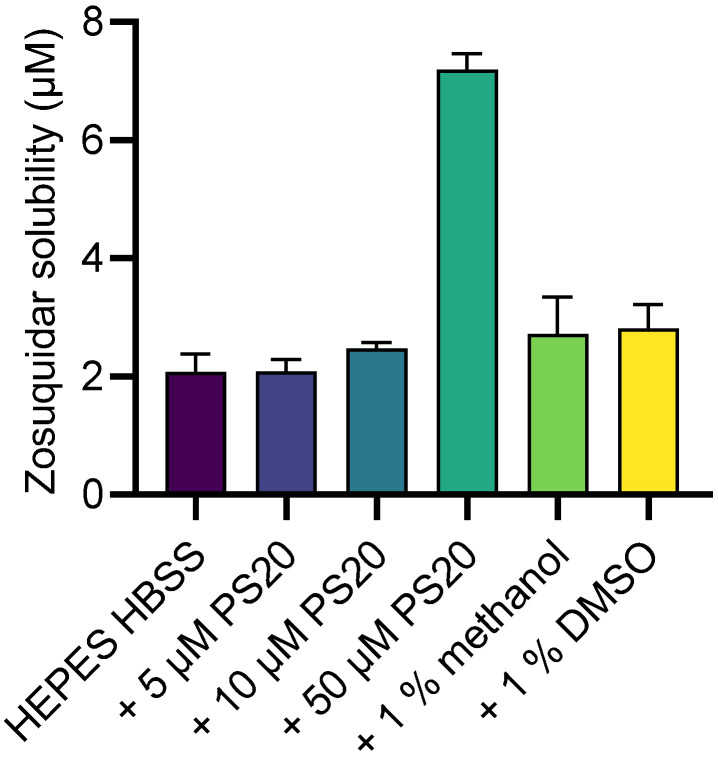
Zosuquidar solubility in 10 mM HEPES HBSS pH 7.4 supplemented with 5, 10, or 50 µM polysorbate 20 (PS20), as well as 1% methanol or DMSO. Shown as mean ± SD, *n* = 4.

Zosuquidar solubility in HEPES HBSS buffer at pH 7.4 was 2.08 ± 0.30 µM. Amounts of 5 or 10 µM polysorbate 20, 1% methanol, or 1% DMSO did not significantly affect this, whereas 50 µM polysorbate 20 increased the solubility to 7.19 ± 0.27 µM (Figure 2). The critical micelle concentration (CMC) of polysorbate 20 has been reported to be 11–42 µM at room temperature [23]. It has been reported that surfactants do not affect drug solubilities markedly below their CMC, but increase the apparent solubility above the CMC value [24], which is in accordance with the data observed for zosuquidar and polysorbate 20 in the present study.

### 3.2. Zosuquidar Adsorbed to Various Lab Equipment

**Figure 3:**
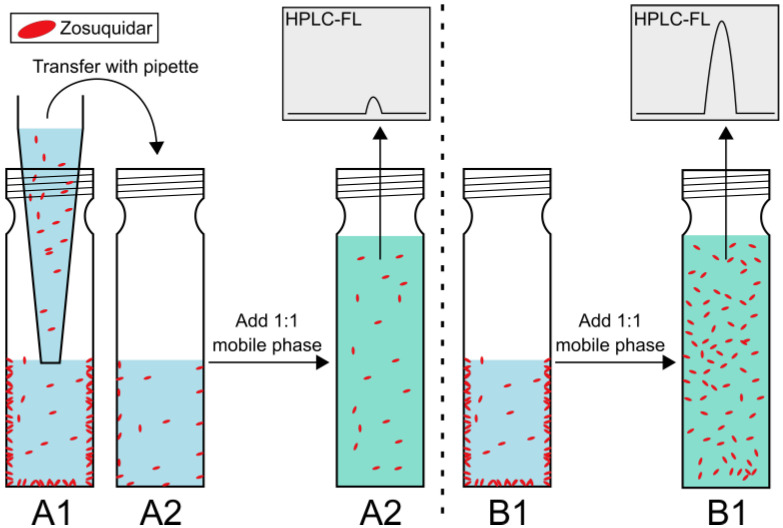
Simple adsorption experiment. Red ellipses symbolise dissolved zosuquidar molecules. Zosuquidar adsorbs to the surfaces of the vial in HEPES HBSS pH 7.4 (blue) but is freely distributed in water–organic solvent mixtures (green). Samples are analysed by fluorescence-coupled HPLC (HPLC-FL). See method section for details. Briefly, two vials, A1 and B1, were filled with HEPES HBSS pH 7.4 and spiked with a concentrated zosuquidar solution. A sample from A1 was transferred to a new vial, A2, and diluted 1:1 in mobile phase. In vial B1, the solution was directly diluted 1:1 in mobile phase in the same vial. The solutions in both vials were then analysed directly by HPLC-FL.

To investigate zosuquidar adsorption to surfaces indicated during the research, a series of experiments were conducted using spiking and serial dilution approaches. Spiking 250 nM zosuquidar to HEPES HBSS pH 7.4 in glass vials resulted in a concentration of 38.1 ± 13.7 nM after transfer to a new vial, and 282 ± 8 nM without transfer (Figure 3). A concentration of 282 ± 8 nM was within a reasonable margin of the theoretical concentration of 250 nM, considering the pipetting of a small volume of pure methanol. Since the working concentrations of 250 nM was well below the equilibrium solubility of zosuquidar, the experiment demonstrated that zosuquidar adsorbed to the surfaces of glass vials in HEPES HBSS pH 7.4 (Figure 3).

**Figure 4:**
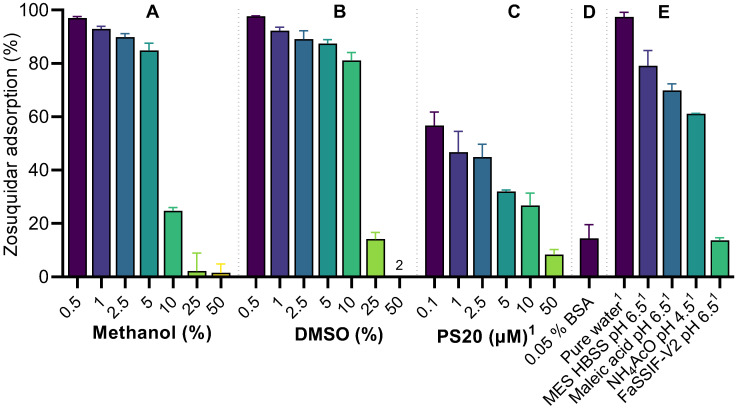
Zosuquidar adsorption (%) in mixtures of methanol (**A**) or DMSO (**B**) in HEPES HBSS pH 7.4, or HEPES HBSS pH 7.4 supplemented with 0.1–50 µM polysorbate 20 (PS20) (**C**) or supplemented with 0.05% bovine serum albumin (BSA) (**D**), or in pure water, 10 mM MES HBSS pH 6.5, 19 mM maleic acid pH 6.5, 10 mM ammonium acetate (NH_4_AcO) pH 4.5, or FaSSIF-V2 pH 6.5 (**E**). Adsorption assessed by addition of 5 µL 100 µM zosuquidar in methanol or DMSO to 995 µL of the relevant buffer for an apparent concentration of 500 nM zosuquidar followed by 24 h equilibration at ambient temperature and analysis of the free zosuquidar concentration by HPLC-FL. ^1^ Final solutions contained 0.5% methanol. ^2^ For 50% DMSO, adsorption was below zero (−5.9 ± 1.1%) and not shown on the graph. Shown as mean ± SD, *n* = 3–4.

The adsorption tendency of zosuquidar in mixtures of water and methanol or DMSO was also assessed (Figure 4A,B). The aim was to estimate the minimal methanol or DMSO concentration that could negate nonspecific zosuquidar adsorption in HEPES HBSS pH 7.4 during in vitro experiments. Zosuquidar adsorption was decreased by increasing concentrations of both methanol and DMSO (Figure 4A,B). At 25% methanol, the nonspecific zosuquidar adsorption was only 2.19 ± 6.71% at a concentration of 500 nM zosuquidar. This was considered acceptable for preparing spiking solutions for subsequent experiments. The maximal amount of zosuquidar that could be dissolved and would not elicit adsorption in 25% methanol in HEPES HBSS pH 7.4 was then investigated by a similar method, where zosuquidar was spiked to achieve a final zosuquidar concentration of 0.75, 2.5, 7.5, or 25 µM in 25% methanol. For 0.75, 2.5, and 7.5 µM zosuquidar, the estimated nonspecific zosuquidar adsorption was 6.83 ± 2.33, 2.76 ± 0.22, and −0.10 ± 2.02%, respectively, whereas the nonspecific zosuquidar adsorption of 25 µM zosuquidar in 25% methanol was 32.5 ± 0.7%. As a result, 7.5 µM zosuquidar in 25% methanol was the maximal zosuquidar concentration that could be used for preparing solutions in HEPES HBSS 7.4 for in vitro experiments.

In HEPES HBSS pH 7.4 + 0.5% methanol, 97.0 ± 0.6% zosuquidar adsorbed to the vial Figure 4A), whereas the presence of polysorbate 20 significantly decreased zosuquidar adsorption in a concentration-dependent manner (Figure 4C).

Zosuquidar adsorption in pure water was high and similar to the adsorption observed in HEPES HBSS pH 7.4 (Figure 4A,E). Lowering the pH to 6.5 in MES HBSS or maleic acid as well as pH 4.5 in ammonium acetate decreased adsorption (Figure 4E). At decreasing pH values, zosuquidar becomes increasingly protonated and positively charged, which may explain the lowered adsorption (Figure 1). Furthermore, zosuquidar adsorption was reduced to 13.6 ± 1.0% in FaSSIF-V2 pH 6.5. FaSSIF-V2, which contains a maleic acid buffer as well as sodium taurocholate (3 mM) and soybean lecithin (0.2 mM). The adsorption in maleic acid buffer and FaSSIF-V2 (Figure 4E) suggest that the presence of surfactants in the form of bile salts and/or phospholipids also significantly contributed to decrease the nonspecific adsorption of zosuquidar.

Adsorption of drug substances to glassware has been described previously, for example, for epinephrine, physostigmine, and atropine [25], amitriptyline and nortriptyline [26], and olanzapine [27], as well as for amitriptyline, atenolol, imipramine, and propranolol [28]. Adsorption of drug substances to glass occurs by either weak cation exchange between silanol groups and basic compounds, or by hydrophobic interactions between siloxane groups and hydrophobic compounds [26,28]. Consequently, adsorption to glass is typically observed for basic hydrophobic compounds, such as zosuquidar. Increasing the ionic strength, lowering the pH of the diluent, or adding organic solvents or surfactants are common strategies to negate adsorption, depending on the expected mechanism of adsorption [26,28]. Except increasing the ionic strength, all these strategies could be applied to decrease zosuquidar adsorption to glass, as demonstrated in the present work.

It was previously reported that zosuquidar tended to adsorb nonspecifically to both plastic and glass surfaces of commonly applied laboratory equipment, when zosuquidar was dissolved in aqueous solutions [5]. In the present work, this phenomenon was systematically investigated in relevant aqueous buffers at various pH values and in the presence of organic solvents or surfactants, and the present work may guide future study design involving aqueous solutions of zosuquidar. Several commonly applied third-generation inhibitors, such as elacridar, tariquidar, and laniquidar, are also hydrophobic and basic compounds with a similar structure to zosuquidar. It may be possible that these compounds elicit similar adsorption to glass, and researchers should be aware of this, when designing experiments. It was outside the scope of the current work to investigate the nonspecific adsorption of other P-gp inhibitors.

### 3.3. Transcellular Etoposide Permeability Studies

**Figure 5:**
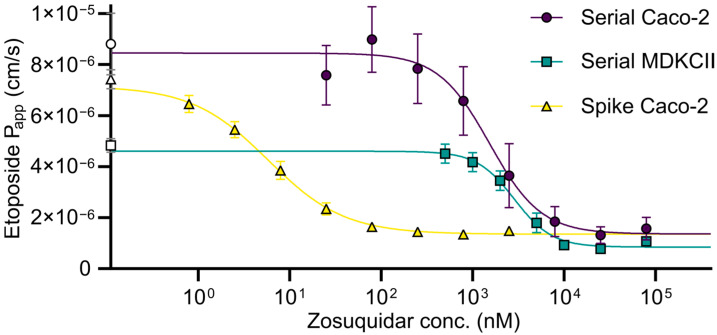
Basolateral-to-apical etoposide permeability across Caco-2 or MDCKII-MDR1 cell monolayers in the presence of 0.79 nM–79 µM zosuquidar, where working solutions are prepared by conventional serial dilution or by spike method (reprinted from [5]). For serially diluted solutions, the plotted concentration is an apparent concentration. Controls without zosuquidar are plotted on the y-axis for comparison (open symbols). Solid lines represent dose–response regression (Equation (1)). Shown as mean ± SEM, *n* = 2–4 (serial) or 4 (spike) experiments in different cell passages in 12-well plates, SEMs smaller than symbol size are not shown.

Before it was discovered that zosuquidar tended to adsorb to surfaces, aqueous solutions for various cell-based in vitro studies of P-gp inhibition were generally prepared by serial dilution of zosuquidar. However, with the finding that zosuquidar did not act ideally in aqueous solutions, the applied protocols were redefined to include a spiking step, where zosuquidar was added directly on a cell compartment from a stock solution in 25–100% methanol. With this method, it was ensured that the added molar amount of zosuquidar in the experiment was known. This spike method was first described for a series of etoposide bidirectional permeability experiments across Caco-2 cell monolayers [5]. In the present work, similar transcellular permeability studies were conducted, where a conventional serial dilution method was applied for the preparation of working solutions (Figure 5), which was then compared to the previously published study that utilised the spike method [5]. Comparison between these studies clearly showed that the preparation method dramatically influences the outcome of the study (Figure 5). In the present study, the estimated IC_50_ value of zosuquidar was 1.53 ± 0.62 and 2.85 ± 0.53 µM in Caco-2 and MDCKII-MDR1 cells, respectively, when solutions were prepared by conventional serial dilution. This was three orders of magnitude higher than the previously reported IC_50_ value of 5.80 ± 1.70 nM [5]. The conventional serial dilution method most likely yielded test solutions that did not contain the desired amount of zosuquidar. Hence, the estimated IC_50_ value was arbitrary. Contrarily, the spike method may offer a more valid estimation of the IC_50_ value.

**Figure 6:**
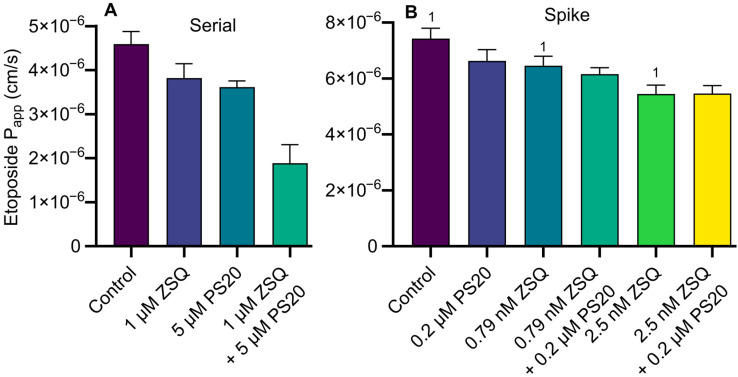
(**A**) Secretory transcellular permeability of 50 µM etoposide across MDCKII-MDR1 cell monolayers in the absence or presence of 1 µM zosuquidar (ZSQ), 5 µM polysorbate 20 (PS20), or a combination of the two (**A**). Test solutions were prepared by a conventional serial dilution method. (**B**) Secretory transcellular permeability of 50 µM etoposide across Caco-2 cell monolayers in the absence or presence of 0.2 µM PS20, 0.79 or 2.5 nM ZSQ or combinations hereof. Zosuquidar added by spike method. Shown as mean ± SEM, *n* = 3 experiments in different cell passages (12-well formats). ^1^ Data from [5].

Likewise, applying a conventional serial dilution method, we observed an apparent additive or even synergistic effect of 1 µM zosuquidar and 5 µM polysorbate 20 on B–A etoposide permeability across MDCKII-MDR1 cell monolayers (Figure 6A). Amounts of 5 µM polysorbate 20 and 1 µM zosuquidar individually decreased the mean etoposide P_app_ to 0.98 and 0.77 × 10^6^ cm/s, whereas 5 µM polysorbate 20 combined with 1 µM zosuquidar decreased P_app_ by 2.71 × 10^6^ cm/s, i.e., 55% more reduction than the effect of the two individual treatments combined (Figure 6A). After changing the protocol to avoid the nonspecific zosuquidar adsorption, the studies were rerun by the spike method (Figure 6B). However, when the spike method was applied, it was observed that there was no or very limited additive effects of the combinations of zosuquidar and polysorbate 20. For instance, 0.2 µM polysorbate 20 decreased B–A etoposide to P_app_ 0.79 × 10^6^ cm/s, compared to control, but there was no difference in mean etoposide P_app_ between 2.5 nM zosuquidar and 2.5 nM zosuquidar combined with 0.2 µM polysorbate 20 (Figure 6B). There was a slight additive effect by combinational inhibition by 0.79 nM zosuquidar and 0.2 µM polysorbate 20 (Figure 6B). This was contrary to the proposed clear synergistic effect observed from the serial dilution method (Figure 6A). Again, the applied method for preparation of test solutions influenced the obtained results. For conventional serial dilutions, the presence of polysorbate 20 in the zosuquidar solutions likely led to more zosuquidar being transferred from one vial to another during serial dilution preparation by decreasing the amount of adsorbed zosuquidar (Figure 4). Thus, initially observed etoposide permeability increased as a result of increased zosuquidar concentrations as an artefact in preparation, rather than from combinational inhibition.

### 3.4. Calcein-AM Assay: Serial Dilution vs. Spike Method and Combinational Inhibition

**Figure 7:**
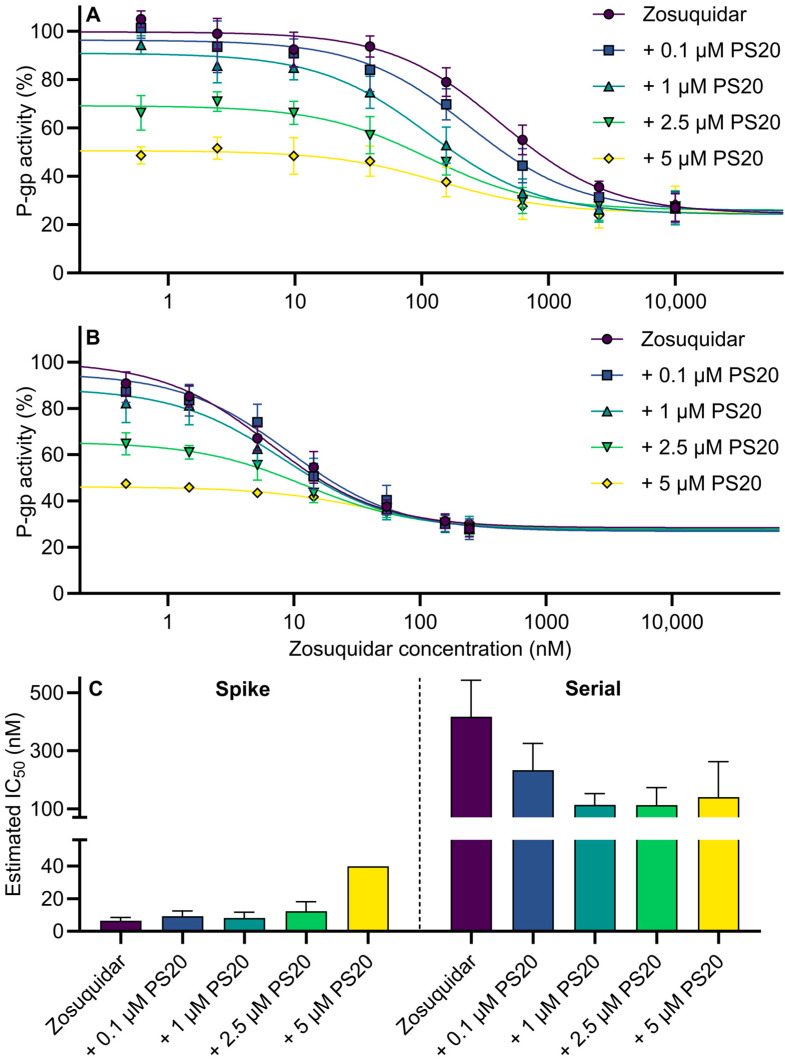
P-gp activity in % as a function of apparent zosuquidar concentration according to calcein-AM assay in the presence of 0, 0.1, 1, 2.5, or 5 µM polysorbate 20 (PS20). Comparison of conventional serial dilution method (**A**) and spike method (**B**) for preparation of test solutions. Resulting IC_50_ values after dose–response regression (Equation (1)) (**C**). Shown as mean ± SEM, *n* = 3–5, except spike method 5 µM PS20, where *n* = 1. *n* represents number of experiments in different cell passages (96-well format). SEMs smaller than the symbol size are not shown.

To verify if the preparation method influenced the transferred amounts of zosuquidar and to assess the effect on P-gp-mediated transport, a calcein-AM assay with zosuquidar and polysorbate 20 was conducted. Similarly, solutions were prepared either by a conventional serial dilution method or by a spike method. These studies were conducted in MDCKII-MDR1 cells parallelly in the same cell passages to compare the two methods head-to-head (Figure 7). Furthermore, the application of a calcein-AM assay increased throughput, which allowed for the combinations of more zosuquidar and polysorbate 20 concentrations, compared to transcellular permeability studies.

For both the conventional serial dilution and the spike method, P-gp activity was decreased by increasing zosuquidar concentrations (Figure 7A,B). Likewise, the presence of polysorbate 20 also decreased P-gp activity, resulting in a shifting of the curves to lower P-gp activity at the lowest zosuquidar concentrations (Figure 7A,B). Considering the combinational P-gp inhibition data produced by the spike method (Figure 7B), there were combinations of zosuquidar and polysorbate 20 concentrations that clearly yielded additive effects. For example, the mean P-gp activity at 1.5 nM zosuquidar was 85% and decreased to 61 and 46% in the presence of 2.5 or 5 µM polysorbate 20, respectively (Figure 7B). This suggested an additive effect of combinational P-gp inhibition by zosuquidar and polysorbate 20. For the conventional serial dilution method, the estimated IC_50_ value of zosuquidar alone was 417 ± 126 nM, whereas the spike method resulted in an estimated IC_50_ value of 6.56 ± 1.92 nM (Figure 7C). Hereby, a 60-fold discrepancy was revealed, simply from applying different methods of test solution preparation, which agreed with findings from transcellular etoposide permeability studies.

The presence of increasing polysorbate concentrations tended to shift the estimated IC_50_ downwards when the conventional serial dilution method was applied (Figure 7A,C). However, application of the spike method showed that polysorbate 20 either did not affect or increased the estimated IC_50_ value (Figure 7B,C). Thus, for the same study, two different methods of media preparation again showed vastly different results, and the spike method most likely represents the most valid method.

Nonspecific zosuquidar adsorption to labware in cell-based assays has, to the best of our knowledge, not been described as a phenomenon before. However, previous studies have shown how serum negates the modulatory effects of some P-gp inhibitors, including common P-gp modulators, quinidine and amiodarone [29,30], and the nonspecific adsorption of hydrophobic compounds in transcellular permeability studies has been discussed in more detail by Ingels and Augustijns [31]. Still, the findings in the present work are relevant for researchers who are working with zosuquidar, or other highly lipophilic compounds with an adsorption tendency. For both in vitro and in vivo studies, this must be considered to ensure that the desired amount of compound is always present when preparing working solutions.

### 3.5. Etoposide Pharmacokinetics

**Figure 8:**
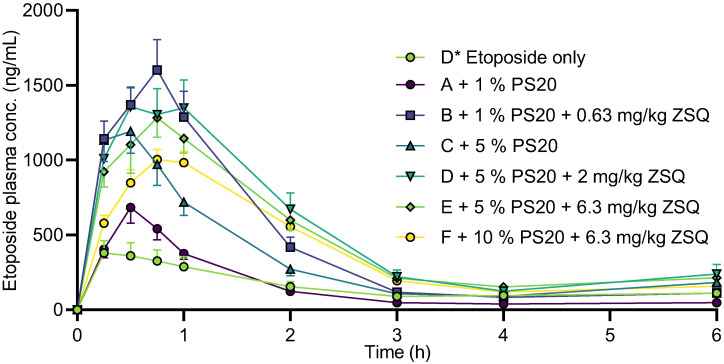
Pharmacokinetic profile of etoposide after oral administration of 20 mg/kg etoposide to male Sprague Dawley rats with coadministration of 1, 5, or 10% polysorbate 20 (PS20) with or without 0.63, 2.0, or 6.3 mg/kg zosuquidar (ZSQ). Starting letter in legends refers to group designation (Table 2). Shown as mean + or—SEM, *n* = 6, SEMs smaller than the symbol size are not shown. Straight connecting lines for illustrative purposes. Groups marked with * represent data from [5].

Zosuquidar and polysorbate 20 showed additive P-gp inhibition in in vitro models that are static models and lack some of the dynamic processes found in vivo. Since zosuquidar and polysorbate 20 have different mechanisms of action on P-gp inhibition, in vivo experiments could provide additional understanding of the possibility of synergistic effects by combined administration on absorption of a P-gp model substrate as etoposide. Zosuquidar and polysorbate 20 were coadministered with etoposide to male Sprague Dawley rats to assess the oral absorption of both zosuquidar and etoposide (Figure 8, Table 2). Results were compared to a previously published study that was conducted at the same facilities in parallel with the present study [5]. Coadministration of 1 and 5% polysorbate 20 altered the pharmacokinetic profile of etoposide (Table 2). For both 1 and 5% polysorbate 20, C_15min_, C_max_, and t_max_ tended to increase. The absolute bioavailability was not affected when 1% polysorbate was coadministered, but it increased from 5.51 ± 0.91% to 11.5 ± 1.4% when 5% polysorbate 20 was coadministered. These observations are in accordance with previous data, where etoposide bioavailability was almost doubled when 5% polysorbate 20 was coadministered relative to etoposide administered alone [7]. In the study by Al-Ali and coworkers, it was also reported that both 5 and 25% polysorbate 20 decreased etoposide bioavailability in P-gp knockout rats, i.e., in an animal model where P-gp-mediated transport is absent. In the study by Al-Ali et al. [7], it was suggested that polysorbate 20 at higher concentrations not only increased absorption by inhibiting P-gp, but also limited full etoposide absorption by retaining etoposide in polysorbate 20 micelles. That a high polysorbate concentration decreased transport was confirmed in a dialysis study, which suggested that reduced free etoposide concentration could be the cause [7]. The reported critical micelle concentration (CMC) of polysorbate 20 in water is in the range of 11–57 μM across temperatures of 22–40 °C [23,32,33,34]. The polysorbate 20 doses at 1 and 5% (*v*/*v*) correspond to 8.9 mM and 44.8 mM, respectively, and thereby, an intestinal dilution of approximately 1000 would be required for the polysorbate 20 concentration to drop below the CMC in the intestine. Assuming a total intestinal fluid volume of 11 mL/kg [35], and considering the administration volume of 10 mL/kg, the dilution was likely closer to a factor of 2.

To directly evaluate if there was an effect from combining polysorbate 20 and zosuquidar on etoposide pharmacokinetics, the dosing groups that were coadministered with 0.63, 2.0, or 6.3 mg/kg zosuquidar [5] were compared with the groups that received combinations of zosuquidar and polysorbate 20, e.g., group F* to B, G* to D, and H* to E and F (Table 2). There was no or very limited effect on pharmacokinetic parameters of etoposide at a low zosuquidar dose at 0.63 mg/kg with or without 1% polysorbate 20. For 2.0 mg/kg zosuquidar with or without 5% polysorbate 20, the presence of polysorbate 20 tended to decrease bioavailability, C_15min_, and C_max_. When 5 or 10% polysorbate 20 was coadministration with 6.3 mg/kg zosuquidar, the bioavailability, C_15min_, and C_max_ all decreased. These findings contradicted any additive or synergistic effect from zosuquidar and polysorbate 20 on the P-gp inhibition in vivo at the administered doses. Instead, it seemed that zosuquidar was the main driver for increasing oral etoposide absorption, whereas polysorbate 20 showed no potentiating effects and even negated the effect of zosuquidar at certain doses.

Many contributing mechanisms could be present in these formulations, where both etoposide, zosuquidar, and polysorbate 20 were administered together, including (i) P-gp modulation by zosuquidar and/or polysorbate 20; (ii) solubilisation of etoposide and/or zosuquidar by polysorbate 20; (iii) retainment of etoposide in polysorbate 20 micelles, which may decrease permeability of etoposide; (iv) retainment of zosuquidar in polysorbate 20 micelles, which may retain zosuquidar from interacting with P-gp. In relation to (iii) and (iv), it should be noted that the observed combinational P-gp inhibition occurred below polysorbate 20’s CMC value in vitro, while in vivo intestinal polysorbate 20 concentrations were well above the CMC value. The lack of additive or synergistic effects of combinational inhibition in vivo is evident, yet the underlying mechanism is likely too complex to allow for a single attribution.

### 3.6. Zosuquidar Pharmacokinetics

**Figure 9:**
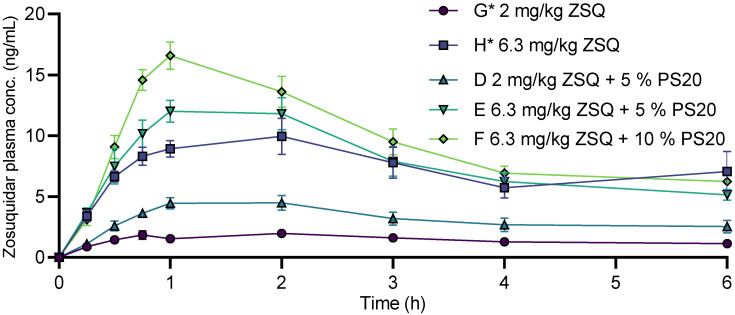
Pharmacokinetic profiles of zosuquidar after oral administration to male Sprague Dawley rats. Coadministered with 20 mg/kg etoposide and 5 or 10% *v/v* polysorbate 20. Starting letter in legends refer to group designation (Table 2). Shown as mean ± SEM, *n* = 6, SEMs smaller than the symbol size are not shown. Straight connecting lines for illustrative purposes. Groups marked with * represent data from [5].

The oral absorption of zosuquidar when coadministered with etoposide and polysorbate 20 was also assessed in the present study (Figure 9). The absolute bioavailability of zosuquidar was reported to be 2.58 ± 0.23% and 4.12 ± 0.51% for 2.0 and 6.3 mg/kg zosuquidar, respectively [5]. For 2 mg/kg zosuquidar, the bioavailability significantly increased to 5.64 ± 0.83% when coadministered with 5% polysorbate 20 in the current study. Similarly, zosuquidar bioavailability at a dose of 6.3 mg/kg tended to increase to 4.45 ± 0.35 or 5.38 ± 0.40% after coadministration with 5 or 10% polysorbate 20, respectively (Table 2). The same pattern was observed for C_max_, where both 5 and 10% polysorbate 20 tended to increase C_max_ of both 2.0 and 6.3 mg/kg zosuquidar (Table 2).

Thus, polysorbate 20 generally increased zosuquidar absorption. Solubilisation of zosuquidar by polysorbate 20 may be the reason; however, the administered etoposide and zosuquidar solutions were stable upon dilution in rat simulated intestinal fluid [5], and therefore, solubilising effects were considered unlikely. The effect of 5% *v/v* polysorbate 20 coadministration on zosuquidar bioavailability was more pronounced at 2.0 mg/kg zosuquidar than at 6.3 mg/kg zosuquidar (Table 2). Elacridar and tariquidar, which are structurally similar to zosuquidar have been reported to be transported by P-gp at low nanomolar concentrations [13,36]. Assuming zosuquidar is also transported by P-gp, polysorbate 20 could increase zosuquidar absorption by modulation of P-gp, especially at low zosuquidar concentrations. The present study was, to our knowledge, the first study to assess oral absorption of a third-generation P-gp inhibitor in the presence of P-gp-inhibiting surfactants.

## 4. Conclusions

The present study showed that zosuquidar elicited nonspecific adsorption to various laboratory materials, and that this adsorption must be mitigated in the preparation of zosuquidar solutions for experiments. Specifically, this study showed how estimated IC_50_ values of zosuquidar on P-gp-mediated transport were vastly different in two different in vitro assays as a result of the different protocols used for preparation of the solutions. This serves as valuable information for researchers who are designing new experiments with zosuquidar and other similar and highly hydrophobic compounds. Zosuquidar and polysorbate 20 elicited additive effects on P-gp-mediated transport in vitro at certain concentrations. Contrarily, the oral absorption of etoposide in vivo was decreased by coadministration of zosuquidar and polysorbate 20, compared to coadministration of zosuquidar alone. A complex interplay between etoposide, zosuquidar, and polysorbate 20 in the intestines with retention of etoposide and zosuquidar in polysorbate 20 micelles may be a contributing mechanism. Furthermore, the coadministration of polysorbate 20 increased oral zosuquidar absorption, which may also have contributed to decrease the effect of zosuquidar on oral etoposide absorption by clearing zosuquidar from the intestinal lumen. Overall, the present work investigated the hypothesis that two different P-gp inhibitors with different inhibitory mechanisms could be combined and thereby reduce the individual doses needed for increasing the oral absorption of a P-gp substrate. For zosuquidar and polysorbate 20 at the investigated doses, this was not the case, though. In this given case, the in vitro results were relatively predictive for the in vivo results, and this adds to implementation of 3R for future similar studies.

## Figures and Tables

**Table 1:** Overview of transcellular permeability studies, series 1–3.

Experiment Series	1	2	3
Method	Conventional serial dilution	Conventional serial dilution	Spike method
Cell line	Caco-2	MDCKII-MDR1	Caco-2
No. of cell passages	4	4	4
Direction of permeation	A–B + B–A	B–A	A–B + B–A
Cell layer integrity	TEER + ^3^H-mannitol permeability (post study)	TEER + Lucifer yellow permeability	TEER + Lucifer yellow permeability

A–B and B–A, apical to basolateral and vice versa; TEER, transepithelial electrical resistance.

**Table 2:** Pharmacokinetic parameters.

	[5]				Present Study					
Group Name	D*	F*	G*	H*	A	B	C	D	E	F
Etoposide dose (mg/kg)	20	20	20	20	20	20	20	20	20	20
Zosuquidar dose (mg/kg)	-	0.63	2.0	6.3	-	0.63	-	2.0	6.3	6.3
Polysorbate 20 conc. (% *v*/*v*)	-	-	-	-	1.0	1.0	5.0	5.0	5.0	10
**Etoposide pharmacokinetics**										
AUC_0–6h_ (µg/mL × h)	0.947 ± 0.156	2.47 ± 0.44	3.86 ± 0.50	4.27 ± 0.47	0.921 ± 0.084	2.61 ± 0.24	1.97 ± 0.24	3.08 ± 0.38	2.80 ± 0.20	2.31 ± 0.16
Bioavailability (%)	5.51 ± 0.91	14.4 ± 2.5	22.4 ± 2.9	24.8 ± 2.7	5.35 ± 0.49	15.2 ± 1.4	11.5 ± 1.4	17.9 ± 2.2	16.3 ± 1.2	13.4 ± 1.0
C_15min_ (µg/mL)	0.381 ± 0.082	1.29 ± 0.16	1.62 ± 0.26	1.69 ± 0.18	0.404 ± 0.058	1.14 ± 0.12	1.13 ± 0.14	1.01 ± 0.10	0.923 ± 0.103	0.579 ± 0.052
C_max_ (µg/mL)	0.390 ± 0.084	1.59 ± 0.29	2.24 ± 0.54	2.11 ± 0.20	0.711 ± 0.086	1.75 ± 0.16	1.22 ± 0.14	1.43 ± 0.18	1.30 ± 0.13	1.02 ± 0.07
t_max_ (min)	15 [15;30]	45 [15;45]	38 [26;60]	45 [15;60]	30 [30;34]	45 [30;49]	30 [26;30]	38 [30;60]	45 [41;49]	45 [41;60]
t_½_ (min)	80.8 ± 13.2	39.3 ± 3.0	51.4 ± 2.4	56.5 ± 4.8	42.4 ± 2.4	37.2 ± 1.2	43.3 ± 2.0	52.9 ± 2.8	54.7 ± 4.5	56.4 ± 2.2
**Zosuquidar pharmacokinetics**										
AUC_0–6h_ (ng/mL × h)		BLQ	8.67 ± 0.77	43.6 ± 5.4		BLQ		18.9 ± 2.8	47.0 ± 3.7	56.8 ± 4.2
Bioavailability (%)			2.58 ± 0.23	4.12 ± 0.51				5.64 ± 0.83	4.45 ± 0.35	5.38 ± 0.40
C_max_ (ng/mL)			2.10 ± 0.27	10.9 ± 1.2				4.8 ± 0.5	13.2 ± 1.0	16.6 ± 1.1
t_max_ (min)			120 [45;135]	90 [45;120]				120 [56;120]	60 [56;120]	60 [56;75]
t_½_ (min)			217 ± 27	161 ± 13				180 ± 23	152 ± 20	147 ± 10

Area under the curve from 0–6 h (AUC_0–6h_), bioavailability, initial plasma concentration at 15 min (C_15min_), maximal plasma concentration (C_max_), and plasma half-life (t_½_) given as mean ± SEM, t_max_ given as median [Q1; Q3], *n* = 6. BLQ = zosuquidar concentration in plasma samples were below LOQ. Pharmacokinetic parameters from a parallelly conducted study [5] reprinted for comparison and denoted with * (groups D*, F*, G*, and H*). Bioavailability of etoposide and zosuquidar was calculated based on IV data from the same study.

## Data Availability

Data available from corresponding author upon request.
